# Quantitative Profiling of Oncometabolites in Frozen and Formalin-Fixed Paraffin-Embedded Tissue Specimens by Liquid Chromatography Coupled with Tandem Mass Spectrometry

**DOI:** 10.1038/s41598-019-47669-5

**Published:** 2019-08-02

**Authors:** Xun Bao, Jianmei Wu, Brian Shuch, Patricia LoRusso, Ranjit S. Bindra, Jing Li

**Affiliations:** 1 Karmanos Cancer Institute, Wayne State University School of Medicine, Detroit, MI 48201 USA; 2 Yale Cancer Center, Yale University School of Medicine, New Haven, CT 06520 USA; 3 Department of Urology, David Geffen School of Medicine at UCLA, Los Angeles, CA 90049 USA

**Keywords:** Liquid chromatography, Biomarkers

## Abstract

Given the implications of oncometabolites [succinate, fumarate, and 2-hydroxyglutarate (2HG)] in cancer pathogenesis and therapeutics, quantitative determination of their tissue levels has significant diagnostic, prognostic, and therapeutic values. Here, we developed and validated a multiplex liquid chromatography coupled with tandem mass spectrometry (LC-MS/MS) platform that allows simultaneous determination of oncometabolites (including succinate, fumarate and total 2HG) and other tricarboxylic acid cycle metabolites (α-ketoglutarate, malic acid, and glutamate) in frozen and FFPE tissues specimens. In addition, by employing chiral derivatization in the sample preparation, the platform enabled separation and determination of 2HG enantiomers (D- and L-2HG) in frozen and FFPE tissues. Isotope-labeled internal standard method was used for the quantitation. Linear calibration curve ranges in aqueous solution were 0.02–10, 0.2–100, 0.002–10, and 0.002–5 µM for succinate, fumarate, total 2HG, and D/L-2HG, respectively. Intra- and inter-day precision and accuracy for individual oncometabolites were within the generally accepted criteria for bioanalytical method validation (<15%). The recovery of spiked individual oncometabolites from pooled homogenate of FFPE or frozen tissue ranged 86–112%. Method validation indicated the technical feasibility, reliability and reproducibility of the platform. Oncometabolites were notably lost during the routine FFPE process. The ratios of succinate to glutamate, fumarate to α-ketoglutarate, 2HG to glutamate, and D-2HG to L-2HG were reliable surrogate measurements for the detection of altered levels of oncometabolites in FFPE specimens. Our study laid a foundation for the utility of archival FFPE specimens for oncometabolite profiling as a valid technique in clinical research and routine medical care.

## Introduction

It has been discovered that abnormal accumulations of some metabolites due to loss- or gain-of-function mutations in genes encoding metabolic enzymes can cause both metabolic and non-metabolic dysregulation and malignancy transformation^1–3^. Given their significant roles in cellular transformation and oncogenesis, these metabolites have been defined as oncometabolites^4^. For example, loss-of-function germline mutations in genes encoding tricarboxylic acid (TCA) cycle enzymes succinate dehydrogenase subunits (SDH) and fumarate hydratase (FH) lead to abnormal accumulation of succinate and fumarate, respectively^5^. By contrary, gain-of-function mutations in genes encoding isocitrate dehydrogenase-1 and -2 (IDH1/2), whose normal function is to catalyze the conversion of isocitrate to α-ketoglutarate (α-KG) in the TCA cycle, confer a neomorphic enzyme activity of the encoded protein that converts α-KG to an oncometabolite, 2-hydroxyglutarate (2HG)^6^. Notably, 2HG, which is a five-carbon dicarboxylic acid with a chiral center at the second carbon atom, is presented as two enantiomers, D-2HG (or known as R-2HG) and L-2HG (or known as S-2HG). While they share identical physicochemical properties, D- and L-2HG have distinct biochemical and biological effects. For example, D-2HG is produced by *IDH1/2* mutations commonly found in gliomas and acute myeloid leukaemia^7,8^; whereas, L-2HG is selectively produced in hypoxic cells and elevated L-2HG levels have been identified in renal cell carcinoma^9,10^. These oncometabolites dysregulate cellular processes through competitive inhibition of α-ketoglutarate–dependent dioxygenases, broad epigenetic changes (e.g., DNA and histones hypermethylation), and other yet unidentified mechanisms^1,2^.

Our research group recently made a seminal discovery that oncometabolites including 2HG, succinate and fumarate potently suppress homologous recombination (HR) DNA repair and render tumor exquisite sensitivity to DNA repair inhibitors such as poly(ADP)-ribose polymerase (PARP) inhibitors^11,12^. These findings uncovered a completely new treatment strategy by exploiting a previously unrecognized HR defects for a variety of tumor types with elevated oncometabolite levels. A number of multi-center clinical trials are ongoing to explore oncometabolites as potential biomarkers for DNA repair defects and tumor sensitivity to PARP inhibitors. Thus, it is imperative that a sensitive, reliable, and reproducible analytical method is developed for quantitative profiling oncometabolites in tumors.

Several mass spectrometry-based methods have been developed for the determination of succinate, fumarate, or 2HG (including total 2HG, D- and L-2HG) in plasma and frozen tissues^13–17^. These methods, however, were limited by either low sensitivity or tedious sample preparation. In addition, no methods have been published for simultaneous determination of fumarate, succinate, and 2HG in formalin-fixed paraffin-embedded (FFPE) tissue specimens although there was one published paper describing the detection of 2HG only in FFPE specimens by gas chromatography-mass spectrometry (GC-MS)^15^. As frozen tumor tissues are not routinely available when evaluating patients for new therapeutic options and often requires a fresh biopsy and immediate processing, the ability to determine oncometabolites in FFPE tissue specimens would be immensely advantageous because of the widespread availability of archival FFPE specimens in routine medical care.

Here, we developed and fully validated a multiplex liquid chromatography coupled with tandem mass spectrometry (LC-MS/MS) platform that allowed simultaneous determination of oncometabolites (including succinate, fumarate, and total 2HG) and other TCA cycle metabolites (e.g., α-ketoglutarate, malic acid, and glutamate) in not only frozen tissues but also FFPE specimens. In addition, by employing chiral derivatization in the sample preparation, the platform enabled separation and determination of 2HG enantiomers (D- and L-2HG) in frozen and FFPE tissues. Method validation indicated the technical feasibility, reliability and reproducibility of the platform. Furthermore, we demonstrated that while oncometabolites (succinate, fumarate, and 2HG) were notably lost during the routine FFPE process, the ratios of succinate to glutamate, fumarate to α-ketoglutarate, and 2HG to glutamate remained consistent between FFPE and matched frozen tissue specimens. Our study laid a foundation for the utility of archival FFPE specimens for oncometabolite profiling as a valid technique in clinical research and routine medical care.

## Results and Discussion

### Workflow of the multiplex LC-MS/MS platform

**Figure 1:**
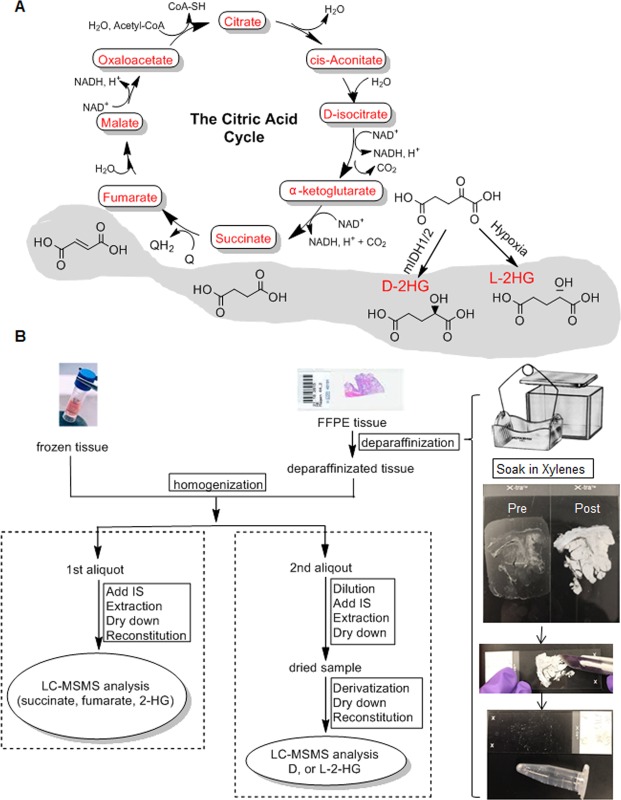
(**A**) Abnormal accumulations of succinate, fumarate, and 2-hydroxyglutarate (2HG), due to loss- or gain-of-function mutations in genes encoding the citric acid cycle enzymes. (**B**) Overall workflow of the multiplex LC-MS/MS platform for simultaneous determination of succinate, fumarate, and total 2HG, as well as D- and L-enantiomers of 2HG in FFPE or frozen tissue specimens.

Figure 1A shows the TCA cycle and chemical structures of oncometabolites (succinate, fumarate, and 2HG), which share a similar structure with the normal TCA metabolite, α-KG. Notably, 2HG carries a chiral carbon atom and occurs in two enantiomers, D- and L-2HG. Given the identical physicochemical properties of 2HG enantiomers, strategies using either chiral column or chiral derivatization was developed for quantitation of D- and L-2HG^17^. The former requires an expensive chiral column and has poor detection sensitivity due to inefficient ionization of D- and L-2HG in the mass spectrometer. Chiral derivatization followed by LC-MS/MS analysis have been shown to significantly improve detection sensitivity^17^. Two derivatization reagents including diacetyl-L-tartaric anhydride (DATAN) and N-(p-toluenesulfonyl)-L-phenylalanyl chloride (TSPC) have been reported for the chiral derivatization of 2HG, each with advantages and disadvantages^16,17^. For example, DATAN derivatives of D- and L-2HG requires a shorter chromatographic separation time than TSPC derivatives (running time, 6 min vs. 40 min), but TSPC derivatives show ~100-fold higher sensitivity than DATAN derivatives^16,17^. Because of limited tissue recovered from FFPE slides and low 2HG levels expected in some specimens (e.g., tissues with wild-type *IDH1/2*), assay sensitivity was our priority. Hence, we chose TSPC derivatization in our study. As compared to the published method^17^, our method was faster (running time, 30 min versus 40 min), while achieving equally good separation and sensitivity.

Figure 1B shows the overall workflow of the multiplex LC-MS/MS platform for determination of succinate, fumarate and total 2HG, as well as D- and L-2HG in frozen and FFPE tissue specimens. Briefly, FFPE tissue specimens were deparaffinized by two washes in xylene and dried at room temperature. Deparaffinized FFPE tissue or frozen tissue was homogenized and divided into two aliquots of tissue homogenate. One aliquot was extracted with methanol followed by reversed-phase liquid chromatographic separation on a Phenomenex Synergi Polar-RP column for simultaneous determination of succinate, fumarate and total 2HG as well as other TCA cycle metabolites (including glutamate, malic acid, and α-ketoglutarate). The other aliquot was subjected to TSPC derivatization followed by reversed-phase liquid chromatographic separation on an Inertsil ODS-3 column for determination of D- and L-2HG. The mass transitions and optimized mass parameters for individual metabolites and respective isotope-labeled internal standards are summarized in Supplementary Table 1.

### Method validation

We fully validated the method for quantitation of oncometabolites (succinate, fumarate, total 2HG, as well as D- and L-2HG) in LC-MS grade water as well as in pooled homogenates of FFPE and frozen tissues by assessing the sensitivity, linearity, intra- and inter-day precision and accuracy, recovery, matrix effect, and stability according to the US Food and Drug Administration Guidance to Bioanalytical Method Validation. In addition, we did partial validation to evaluate the precision and accuracy for quantitation of other TCA cycle metabolites (i.e., glutamate, malic acid, and α-ketoglutarate) in water and in pooled homogenates of FFPE and frozen tissues.

#### Sensitivity

**Figure 2:**
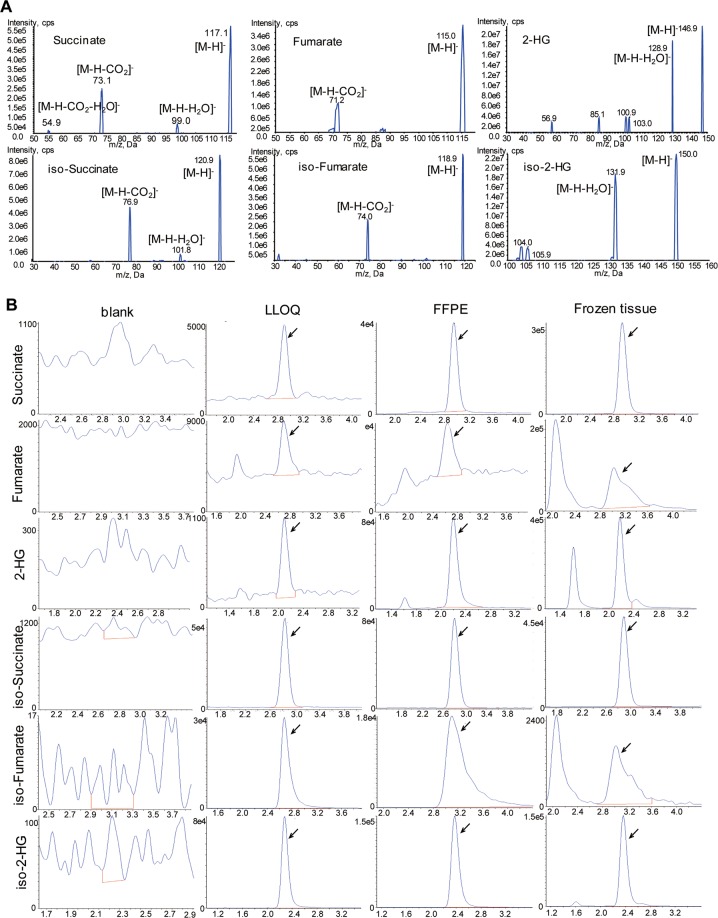
LC-MS/MS analysis of succinate, fumarate, and 2HG. (**A**) MS/MS spectra of succinate, fumarate, and 2HG, as well as their respective isotope-labeled internal standards. (**B**) Extracted chromatograms of succinate, fumarate, and 2HG, as well as their respective isotope-labeled internal standards in the blank (LC-MS grade water), spiked at the LLOQs in water, and the extract from pooled homogenate of FFPE or frozen tissue.

Under the optimized MRM conditions, succinate, fumarate, and total 2HG showed the specific and most sensitive transitions at m/z 117.0 > 73.0, 114.9 > 71.0, and 147.0 > 128.9, respectively (Fig. 2A). They were well retained and separated on a Phenomenex Synergi Polar-RP column under a gradient elution with the overall running time of 13 min (Fig. 2B). The lower limit of quantitation (LLOQ) for succinate, fumarate, and 2HG was determined at 0.02, 0.2, and 0.002 µM, respectively, in aqueous solution (i.e., LC-MS grade water). Figure 2B shows representative chromatograms of succinate, fumarate, and 2HG in the blank (LC-MS grade water), spiked at the LLOQs in water, and the extract from pooled homogenates of FFPE or frozen tissues.

**Figure 3:**
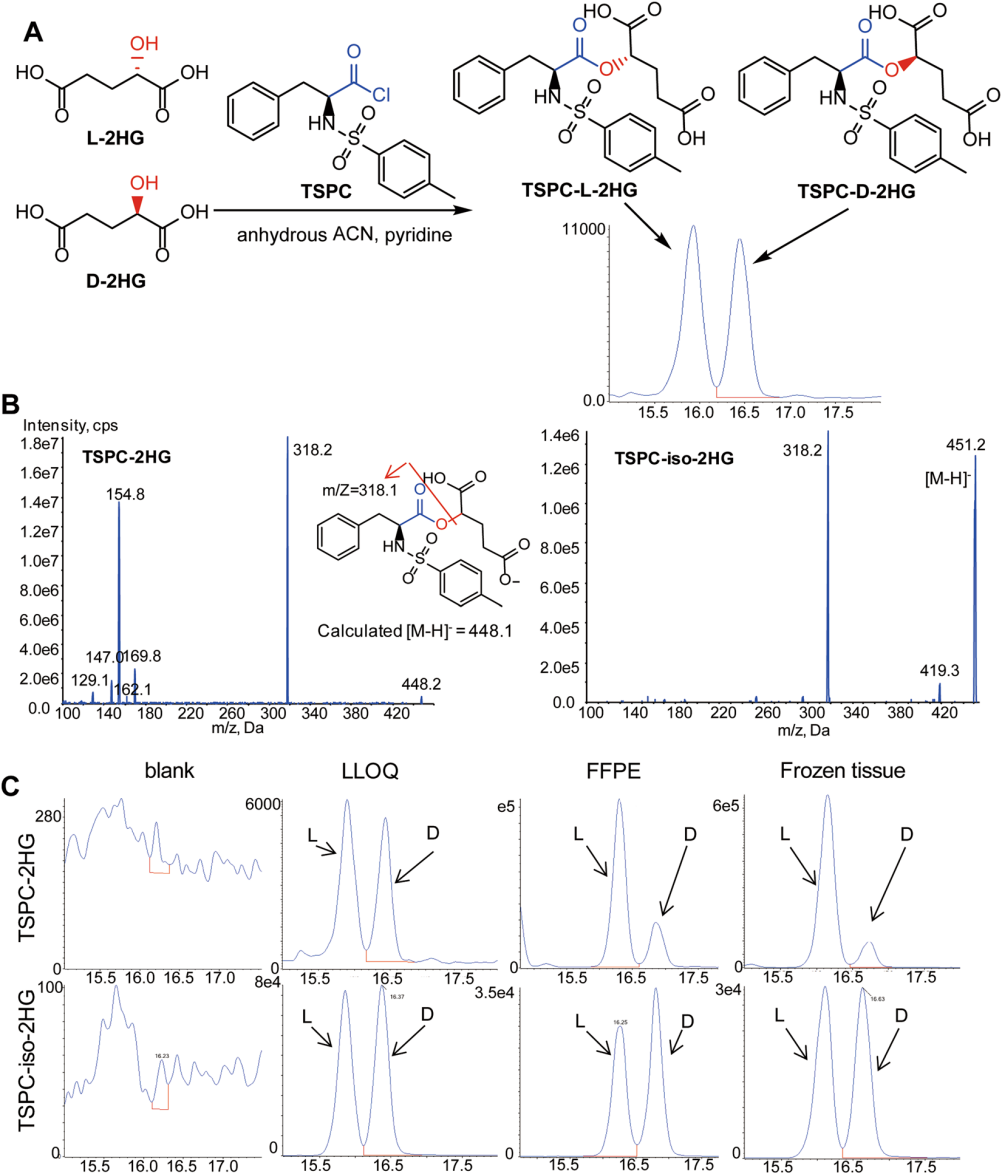
Chiral derivatization followed by LC-MS/MS analysis for determination of D- and L-enantiomers of 2HG. (**A**) Schematic illustration of derivatization of D- and L-2HG by N-(p-toluenesulfonyl)-L-phenylalanyl chloride (TSPC) followed by chromatographic separation of D- and L-2HG TSPC derivatives. (**B**) MS/MS spectra of the TSPC derivatives of D- and L-2HG as well as isotope-labeled D- and L-2HG. (**C**) Extracted chromatograms of TSPC derivatives of D- and L-2HG as well as their respective isotope-labeled internal standards in the blank (LC-MS grade water), spiked at the LLOQs in water, and the extract from pooled homogenate of FFPE or frozen tissue.

TSPC derivatives of D- and L-2HG showed the specific and most sensitive transitions at m/z 448.2 > 318.2 at the optimized MS conditions (Fig. 3A,B). They were adequately separated (with the retention time of 16.5 and 16.2, respectively) on an Inertsil ODS-3 column (250 mm × 2.0 mm i.d., 5 μm, Tokyo, Japan) under a gradient elution (Fig. 3C). The LLOQ for D- and L-2HG was determined at 0.002 µM in aqueous solution (i.e., LC-MS grade water). Figure 3C shows the extracted chromatograms of TSPC derivatives of D- and L-2HG and their respective isotope-labeled internal standards in the blank (LC-MS grade water), spiked at the LLOQs in water, and the extract from pooled homogenates of FFPE or frozen tissues.

#### Linearity, accuracy and precision

Since oncometabolites are endogenously presented in frozen or FFPE samples, we prepare calibration curves in aqueous solution with the stable isotope-labeled internal standard method for quantitation. As the analyte and its stable isotope-labeled analog share similar chromatographic behavior and MS response, the isotope-labeled internal standard method allows correcting for variations in the matrix effect between the calibrators and biological samples^18^.

Linear calibration curves for succinate, fumarate, and total 2HG were established in water at the concentration ranges of 0.02–10, 0.2–100, and 0.002–10 µM, respectively. For 2HG enantiomers, the linear calibration curve was established in water at D- and L-2HG concentration range of 0.002–5 µM. Linear correlation coefficient (R^2^) > 0.99 was obtained in all analytical runs. For all calibrator standards and quality control (QC) samples of individual oncometabolites prepared in water, the intra- and inter-day precision (as assessed by coefficient variation) and accuracy (as assessed by relative estimation error) were within the generally accepted criteria for bioanalytical method validation (<20% at LLOQ and <15% at other QCs) (Table 1 and Supplementary Tables 2, 3). The reproducibility of the method for quantitation of oncometabolites was further demonstrated by the coefficient variations <15% for 6 replicate measurements of baseline and spiked individual oncometabolites in pooled homogenates of FFPE or frozen tissues (Table 2).

**Table 1:** Intra- and inter-day precision and accuracy for the quality control (QC) samples of individual oncometabolites, prepared in LC-MS grade water.

Nominal concentration (µM)	Determined concentration^a^ (µM)	Average accuracy^c^ (%)	Intra-day precision (%)	Inter-day Precision (%)
**Succinate**				
0.02 (LLOQ)	0.021 ± 0.002	9.8	11	11
0.06	0.060 ± 0.006	4.0	7.9	7.1
1.2	1.263 ± 0.073	8.7	2.4	6.1
6.0	6.556 ± 0.160	9.5	2.6	—^b^
**Fumarate**				
0.2 (LLOQ)	0.20 ± 0.02	1.4	8.7	4.4
0.6	0.63 ± 0.04	7.3	5.6	3.4
12	11.35 ± 0.82	−3.9	4.5	6.5
60	60.37 ± 2.39	1.5	3.6	1.9
**Total 2**-**HG**				
0.002 (LLOQ)	0.0020 ± 0.0002	0.0	12	—^b^
0.006	0.0062 ± 0.0004	4.9	5.7	3.8
1.2	1.2647 ± 0.0478	6.0	3.5	1.7
6.0	6.2269 ± 0.2039	3.8	3.5	—^b^
**D**-**2HG**				
0.002(LLOQ)	0.0022 ± 0.0005	10	8.4	—^b^
0.006	0.0062 ± 0.0010	0.6	8.6	5.5
0.8	0.8169 ± 0.0495	2.1	4.8	4.4
4	4.0068 ± 0.2606	0.2	6.3	2.1
**L**-**2HG**				
0.002(LLOQ)	0.0020 ± 0.0003	2.0	13	4.9
0.006	0.0058 ± 0.0006	−3.1	9.1	4.9
0.8	0.7718 ± 0.0509	−3.5	5.5	4.3
4	3.8106 ± 0.1402	−4.7	3.8	—^b^

^a^Each QC was evaluated in quintuplicate on three different days.

^b^One-way analysis of variance: no additional variation was observed as a result of performing assay in different days.

^c^Accuracy was assessed as the relative estimation error of the determined concentration to nominal concentration.

**Table 2:** Recovery of spiked oncometabolites from pooled homogenates of FFPE or frozen tissues.

FFPE	Measured conc. (µM), Mean (SD)	Recovery^a^ (%)	CV^b^ (%)	Frozen Tissue	Measured conc. (µM), Mean (SD)	Recovery^a^ (%)	CV^b^ (%)
**Succinate added** (**µM**)				**Succinate added** (**µM**)			
0	1.580 (0.031)			0	2.192 (0.142)		
LQC (0.5)	2.011 (0.027)	86	6.2	LQC (0.6)	2.710 (0.073)	86	14
HQC (4.0)	6.031(0.132)	108	5.6	HQC (6)	7.988 (0.413)	95	7.2
**Fumarate added** (**µM**)				**Fumarate added** (**µM**)			
0	0.16 (0.006)			0	1.91 (0.17)		
LQC (0.6)	0.79 (0.02)	104	3.9	LQC (0.6)	2.46 (0.10)	96	14
HQC (8)	8.16 (0.28)	100	3.4	HQC (60)	60.14 (2.68)	97	4.6
**Total 2**-**HG added** (**µM**)				**Total 2**-**HG added** (**µM**)			
0	0.1170 (0.0025)			0	0.4822 (0.0183)		
LQC (0.04)	0.1618 (0.0027)	112	6.0	LQC (0.12)	0.6023 (0.0152)	100	13
HQC (4.0)	4.5932 (0.1501)	111	3.9	HQC (6.0)	5.9174 (0.2259)	91	4.2
**D**-**2HG added** (**µM**)				**D**-**2HG added** (**µM**)			
0	0.0371(0.0028)			0	0.1101(0.0075)		
LQC (0.01)	0.0101(0.0010)	101%	10%	LQC (0.03)	0.0283(0.0042)	94%	14%
HQC (4)	3.9581(0.1031)	99%	2.6%	HQC (4)	3.8344(0.0456)	96%	1.2%
**L**-**2HG added** (**µM**)				**L**-**2HG added** (**µM**)			
0	0.1167(0.0031)			0	0.5697(0.0312)		
LQC (0.03)	0.0292(0.0023)	97%	8.0%	LQC (0.2)	0.1927(0.0248)	96%	13%
HQC (4)	3.7160(0.0767)	93%	2.1%	HQC (4)	3.5549(0.1802)	89%	5.1%

^a^Individual oncometabolites were spiked at low (i.e., ~30% of endogenous level) and high (i.e., high QC) concentrations in pooled homogenates of FFPE or frozen tissues. Recovery = (measured total concentration − measured baseline concentration)/nominal spiked concentration.

^b^Coefficient variation from 6 replicate measurements.

For other TCA cycle metabolites, linear calibration curve ranges were 0.02–2 µM, 0.02–10 µM, and 0.2–100 µM in water for glutamate, malic acid, and α-ketoglutarate, respectively. Linear correlation coefficient (R^2^) > 0.99 was obtained in all analytical runs. For all calibrator standards of individual TCA cycle metabolites prepared in water, the intra- and inter-day precision and accuracy were <15% (Supplementary Table 4). The reproducibility for quantitation of TCA metabolites was further demonstrated by the intra- and inter-day coefficient variations <15% from the measurements on three different days with 5 replicates on each day for individual metabolite baseline levels in pooled homogenates of FFPE or frozen tissues (Supplementary Table 5).

Table 2 shows the recovery of spiked individual oncometabolites in pooled homogenates of FFPE or frozen tissues, which was calculated as (measured total concentration − measured baseline concentration)/nominal spiked concentration. When spiked at low (i.e., ~30% of endogenous level) and high concentrations (i.e., high QC level) into pooled homogenates (n = 6), the mean recovery of individual oncometabolites were within 86–112% and 86–100% in pooled homogenates of FFPE and frozen tissue, respectively, and the coefficients variation of 6 replicate measurements were <15%.

#### Matrix effect

Since oncometabolites are naturally presented in FFPE or frozen tissues, their respective isotope-labeled analogs were used for the assessment of the matrix effects on oncometabolites. In pooled FFPE tissue homogenates, the average matrix factor (n = 6) was 0.95, 0.98, and 1.04 for succinate-d6, fumarate-1,4-^13^C2, 2,3-d2 and (RS)-2-hydroxyglutarate-2,3,3-d3, respectively. In pooled frozen tissue homogenates, the average matrix factor (n = 6) was 0.94, 0.43, and 1.18 for succinate-d6, fumarate-1,4-^13^C2, 2,3-d2, and (RS)-2-hydroxyglutarate-2,3,3-d3, respectively, suggesting tissue homogenate had significant matrix effect on fumarate but not on succinate or 2HG. Significant matrix effect of frozen tissue homogenates on fumarate was also indicated by peak shape distortion of fumarate in frozen tissue samples as compared to neat solution samples (Fig. 2B). Similarly, FFPE or frozen tissue homogenates exhibited significant matrix effect on TSPC derivatives of D- and L-2HG. Specifically, the average matric factor for the TSPC derivative of D-2HG was 0.61 and 0.45 in the pooled homogenates of FFPE and frozen tissue, respectively, with the coefficient variations <13% (n = 7). The average matric factor for the TSPC derivative of L-2HG was 0.52 and 0.44 in the pooled homogenates of FFPE and frozen tissue, respectively, with the coefficient variations <14% (n = 7). Given the matrix effect on some oncometabolites, it is critical to use stable isotope-labeled internal standard method for accurate quantitation of oncometabolites in biological samples.

#### Stability

The stability data of succinate, fumarate, and 2HG are summarized in Table 3. Bench-top stability tests at room temperature suggested that three oncometabolites were stable in aqueous solution (water) at 1 or 100 µM for at least 4 hours. Autosampler stability test showed that all 3 oncometabolites were stable in both solution (water) and FFPE or frozen tissue extracts at 4°C for at least 15 h (Table 3), suggesting an analytical run can be performed continuously overnight for a large batch of samples. Freeze-thaw stability test indicated no apparent degradation of all oncometabolites in FFPE or frozen tissue homogenate after three freeze-thaw cycles (Table 3).

**Table 3:** Stability tests for succinate, fumarate, and 2-HG.

	Succinate		Fumarate		2-HG	
Bench-top stability in water (25 °C) (%)^a^	1 µM	100 µM	1 µM	100 µM	1 µM	100 µM
2.0 h	104	98	97	96	104	96
4.0 h	104	102	99	106	104.	102
Bench-top stability in FFPE tissue homogenate (25 °C) (%)^a^	LQC^c^	HQC^c^	LQC^c^	HQC^c^	LQC^c^	HQC^c^
2.0 h	97	98	94	97	103	97
4.0 h	98	101	89	103	103	100
Bench-top stability in frozen tissue homogenate (25 °C) (%)^a^	LQC^c^	HQC^c^	LQC^c^	HQC^c^	LQC^c^	HQC^c^
2.0 h	67	79	95	91	101	92
4.0 h	57	66	102	104	111	97
Bench-top stability in FFPE tissue homogenate (keep on ice) (%)^a^	LQC^c^	HQC^c^	LQC^c^	HQC^c^	LQC^c^	HQC^c^
2.0 h	97	98	94	97	99	103
4.0 h	98	101	89	103	104	102
Bench-top stability in frozen tissue homogenate (keep on ice) (%)^a^	LQC^c^	HQC^c^	LQC^c^	HQC^c^	LQC^c^	HQC^c^
2.0 h	85	88	95	91	101	92
4.0 h	81	91	102	104	111	97
Auto-sampler stability (in LC-MS grade water) (4 °C) (%)^a^	LQC^c^	HQC^c^	LQC^c^	HQC^c^	LQC^c^	HQC^c^
3.0 h	104	103	107	102	87	96
9.0 h	100	101	97	102	95	99
15.0 h	98	103	99	103	89	98
Auto-sampler stability in FFPE tissue extract (4 °C) (%)^a^	LQC^c^	HQC^c^	LQC^c^	HQC^c^	LQC^c^	HQC^c^
3.0 h	98	101	101	98	98	101
9.0 h	98	99	96	98	102	102
15.0 h	100	101	99	97	100	103
Auto-sampler stability in frozen tissue extract (4 °C) (%)^a^	LQC^c^	HQC^c^	LQC^c^	HQC^c^	LQC^c^	HQC^c^
3.0 h	97	100	94	98	88	102
9.0 h	100	99	99	96	91	102
15.0 h	98	101	95	94	89	101
Freeze-thaw stability of FFPE tissue homogenate (−80 °C) (%)^b^	LQC^c^	HQC^c^	LQC^c^	HQC^c^	LQC^c^	HQC^c^
Cycle 1	91	96	90	100	89	98
Cycle 2	91	102	105	104	101	99
Cycle 3	86	99	84	104	88	98
Freeze-thaw stability of frozen tissue homogenate (−80 °C) (%)^b^	LQC^c^	HQC^c^	LQC^c^	HQC^c^	LQC^c^	HQC^c^
Cycle 1	95	97	98	108	106	97
Cycle 2	99	96	93	114	108	99
Cycle 3	98	96	104	101	113	94

^a^Stability data is expressed as the mean percentage of the response (ratio of analyte peak area to isotope labeled internal standard) determined at certain time relative to that at time zero. Each concentration at each time point was assessed in triplicate.

^b^Stability data is expressed as the mean percentage of the analyte concentration determined at certain time point relative to the nominal concentration (%). Each concentration at each time point was assessed in triplicate.

^c^LQC is 0.06, 0.6 and 0.006 µM of succinate, fumarate and 2-HG spiked in homogenate of FFPE or frozen tissue, respectively; HQC is 6, 60, and 6 µM of succinate, fumarate and 2-HG spiked in homogenate of FFPE or frozen tissue, respectively.

Notably, apparent degradation was observed for succinate, and possibly α-ketoglutarate due to their similar properties, in frozen tissue homogenates at room temperature. Further tests by keeping FFPE and frozen tissue homogenates on ice and preventing from light showed that fumarate and 2HG were stable in both FFPE and frozen tissue homogenates for at least 4 hours, while succinate was stable at least 4 hours in FFPE homogenate but only for 2 hours in frozen tissue homogenate (Table 3). Therefore, to minimize potential degradation of oncometabolites and other TCA metabolites during sample preparation, FFPE or frozen tissue homogenates were kept on ice, protected from light, and processed within 2 hours in all subsequent sample preparation and analyses. In addition, stable isotope-labeled internal standards for individual metabolites were included in the sample preparation, which could effectively correct for any potential loss or degradation of the analytes during sample preparation.

### Applications

#### Screening FFPE tumor specimens for elevated D- or L-2HG levels

**Figure 4:**
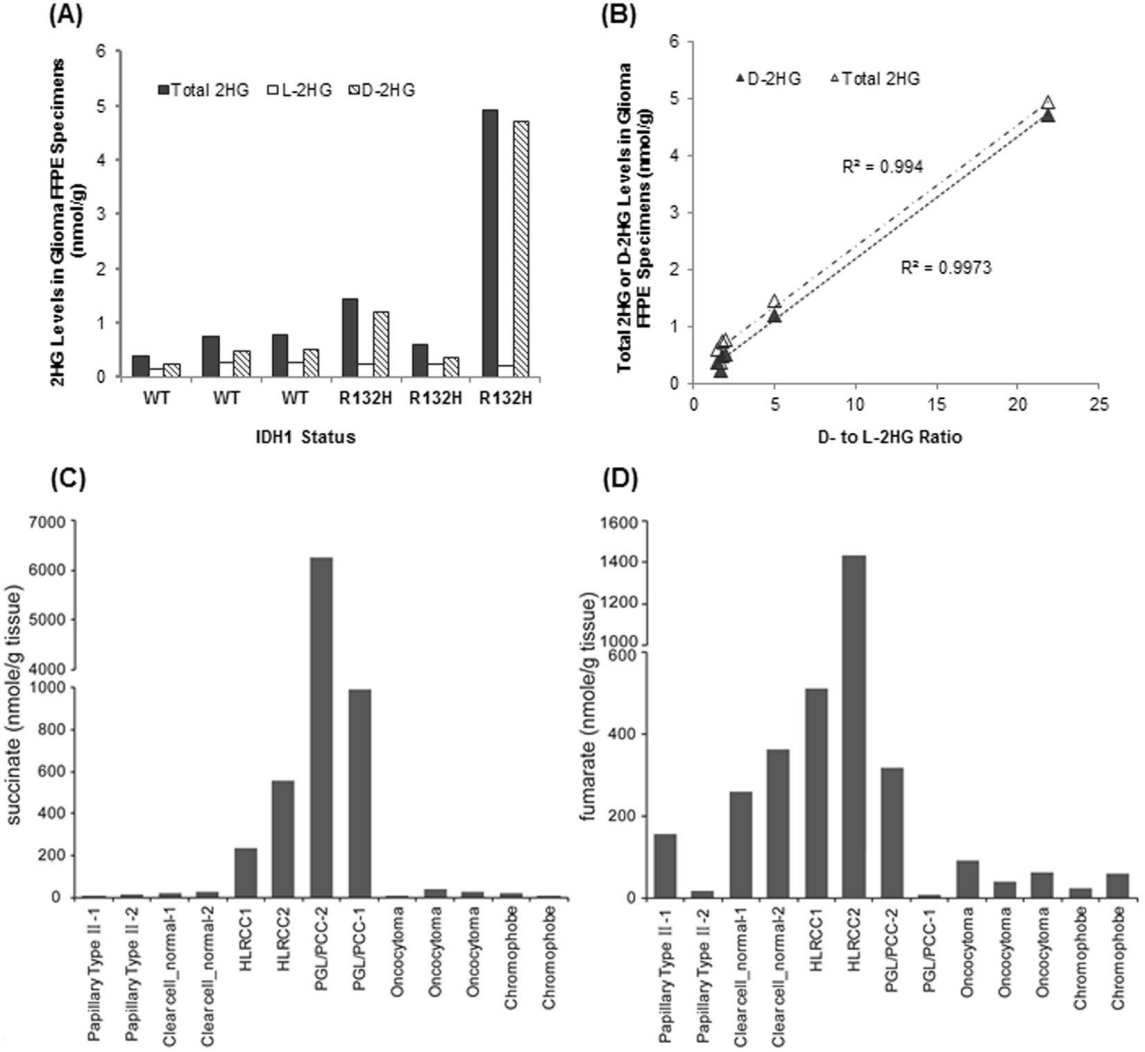
Application examples. (**A**) Detection of elevated levels of 2HG (specifically D-2HG) in glioma FFPE specimens with known IDH1/2 mutations. (**B**) Correlation between D- to L-2HG ratios and total 2HG or D-2HG levels in glioma FFPE specimens. (**C**,**D**) Identification of subsets of hereditary or sporadic renal cell carcinoma frozen tissue samples with overproduction of fumarate or succinate. HLRCC, Hereditary Leiomyomatosis and Renal Cell Cancer (HLRCC); PGL/PCC, Hereditary Paraganglioma – Pheochromocytoma (PGL/PCC).

We discovered that mutant *IDH1/2*-induced 2HG confers HR deficiency, leading to exquisite PARP inhibitor sensitivity^11^. Therefore, measurement of 2HG levels in tumor specimens may provide a potential biomarker for selection of patients for PARP inhibitor treatment. As a proof-of-concept, we determined the levels of total 2HG as well as D- and L-enantiomers of 2HG in 6 glioma FFPE specimens with known *IDH1*/2 status. In accordance with the published data, we found that D-2HG levels, but not L-2HG, were significantly increased in the *IDH1 R132H*-mutated glioma specimens as compared to the *IDH* wild-type specimens (Fig. 4A). Notably, the ratios of D- to L-2HG were highly correlated with total 2HG or D-2HG levels, suggesting D-/L-2HG ratio could be used as the surrogate measurement for detection of elevated 2HG levels, in particular D-2HG, in *IDH1 R132H*-mutated gliomas (Fig. 4B).

#### Identification of subsets of renal cell carcinoma with overproduction of oncometabolites

Our group recently discovered that the abnormal accumulation of succinate and fumarate induced a HR defect, leading to exquisite PARP inhibitor sensitivity^12^. To identify subsets of cancer producing oncometabolites at levels sufficient to confer an HR defect, we determined oncometabolite levels in a collection of hereditary and sporadic renal cell carcinoma frozen tumor tissue samples, using our LC-MS/MS platform. We identified abnormally high accumulation of fumarate and succinate in the specimens derived from Hereditary Leiomyomatosis and Renal Cell Cancer (HLRCC) and Hereditary Paraganglioma – Pheochromocytoma (PGL/PCC), respectively (Fig. 4C,D). These cancer types are known genetic cancer characterized by the heterozygous inheritance of loss-of-function mutations in the *FH* and several *SDH* genes (*SDHA*, *SDHB*, *SDHC*, *SDHD*, and *SDHAF2*), respectively^19,20^. Our data are consistent with the notion that loss-of-function germline mutations in the *FH* and *SDH* genes can result in abnormal accumulation of succinate and fumarate, respectively^5^. We further confirmed HR deficiency specifically in these tumor samples with elevated levels of succinate or fumarate, using the comet assay as the measure of oncometabolite-induced HR defects^12^. These data support that the measurement of succinate and fumarate levels in frozen renal cell carcinoma tumor tissue samples could assist the selection of patients for PARP inhibitor clinical trials to assess therapeutic benefit with the oncometabolite phenotype.

**Figure 5:**
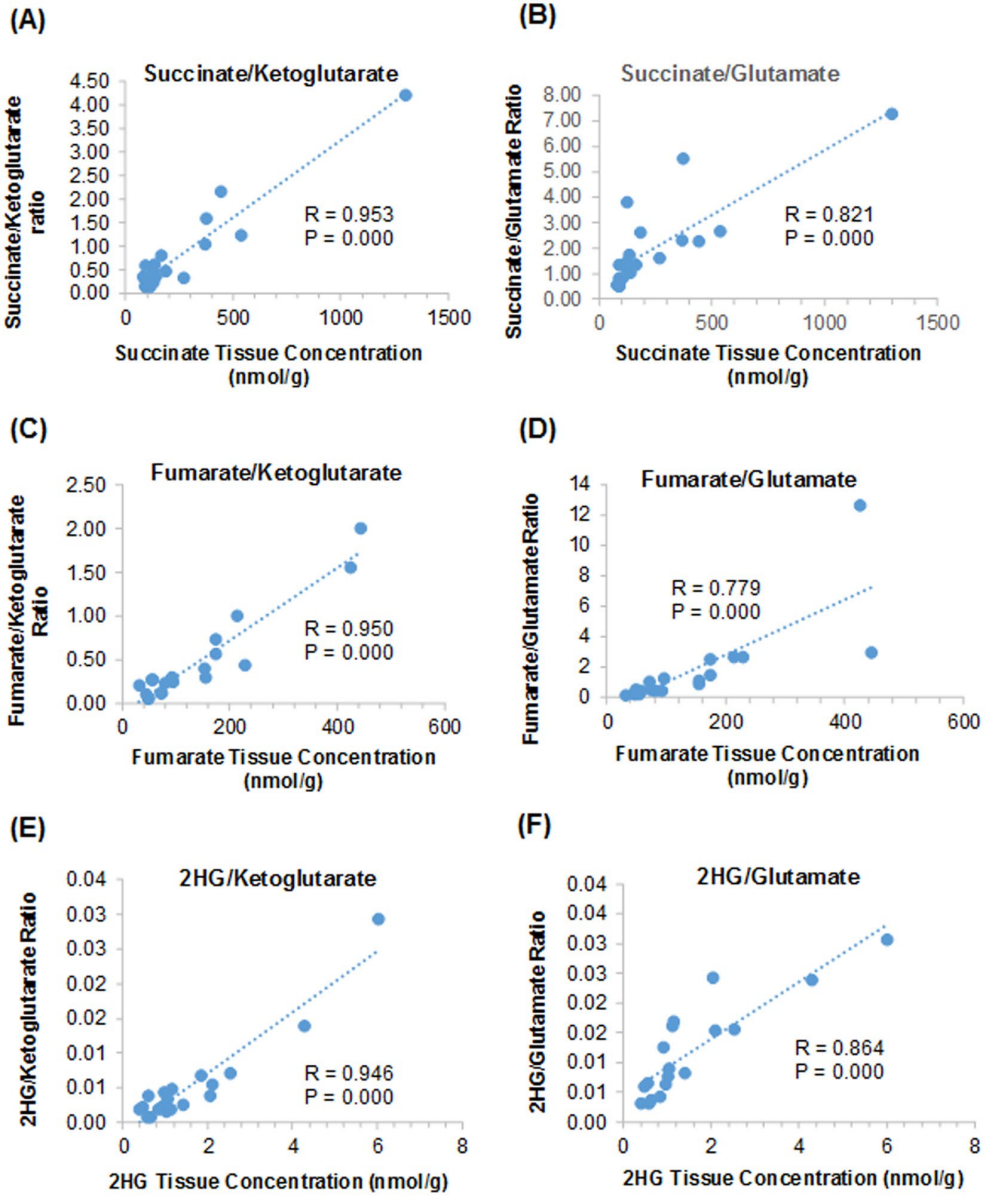
Correlations between individual oncometabolite tissue levels and ratios of oncometabolite to α-ketoglutarate or to glutamate in frozen human renal cell carcinoma and normal kidney tissues.

Of note, while our method validation was focused on oncometabolites (succinate, fumarate, and 2HG) only, our method allowed simultaneous determination of oncometabolites and other TCA cycle metabolites (including α-ketoglutarate, glutamate, and malic acid). Interestingly, we found that individual oncometabolite tissue levels were highly correlated with the ratios of oncometabolite to α-ketoglutarate or to glutamate in frozen tissues (R > 0.8, P < 0.001) (Fig. 5). These data suggested that the ratios of oncometabolite to α-ketoglutarate or to glutamate could be used as the surrogates to detect elevated oncometabolite tissue levels.

### Loss of oncometabolites during FFPE processes

A caveat to screen archival FFPE tissue for oncometabolite levels is the loss of oncometabolites during routine FFPE process, which involves multiple steps including tissue fixation in 10% formalin solution, dehydration in ethanol followed by toluene, and finally embedding in liquid paraffin. Polar, water-soluble metabolites (such as succinate, fumarate, and 2HG) may dissolve in formalin and ethanol solutions, while less polar, lipophilic metabolites may dissolve in ethanol and toluene solvents.

**Figure 6:**
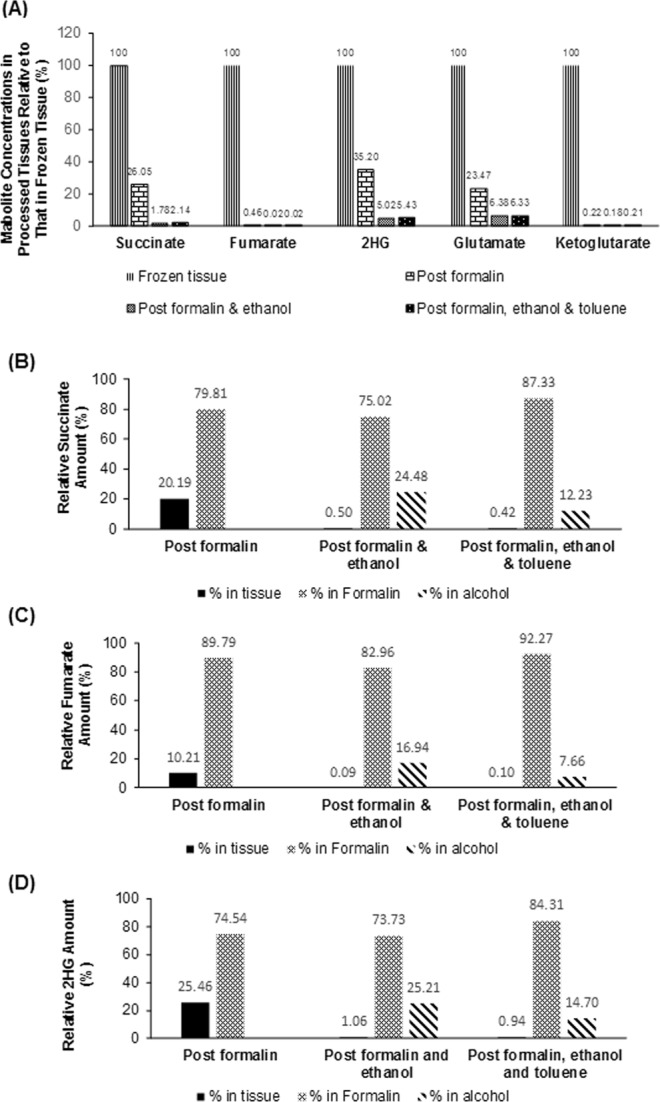
Loss of oncometabolites during multi-step FFPE process. (**A**) Preserved metabolite levels in tissues following formalin-fixation and ethanol-dehydration steps, as the percentage of their respective levels in frozen tissues. (**B**–**D**) The recovery of individual oncometabolites (including succinate, fumarate, and 2HG) in tissues, formalin and ethanol solutions after the fixation and dehydration process. Data are expressed as the ratio of amount recovered in tissue, formalin, or ethanol solution relative to the total amount recovered from both tissue and solutions.

To evaluate the extent of oncometabolite loss during FFPE process, we compared the oncometabolite concentrations in tissues undergoing step-wise FFPE process with those in matched frozen tissues, and additionally we determined oncometabolites that were released into formalin solution, ethanol, and toluene during the fixation and dehydration process. As shown in Fig. 6A, following formalin fixation, preserved tissue concentrations (normalized to tissue weight) of succinate, fumarate, and 2HG were 26.05%, 0.46%, and 35.20% relative to their respective concentrations in matched frozen tissues. Further dehydration in ethanol reduced tissue concentrations of succinate, fumarate, and 2HG to 1.78%, 0.02%, and 5.02% of their respective frozen tissue concentrations. Washing with toluene caused negligible oncometabolite loss. In line with markedly reduced oncometabolite levels in FFPE tissues, approximately 70–90% and 7–25% of oncometabolites were recovered in formalin solution and ethanol, respectively, during the fixation and dehydration process (Fig. 6B–D). Consistent with published data^15^, our data suggested that the major loss of these polar, water soluble oncometabolites (including succinate, fumarate, and 2HG) occurred during formalin fixation and ethanol dehydration process. In addition to the oncometabolites, we also measured the concentrations of other TCA cycle metabolites (α-ketoglutarate, glutamate, and malic acid). Interestingly, the preservation pattern of glutamate in post-fixation and post-dehydration tissues was similar to that of succinate and 2HG, while the preservation pattern of α-ketoglutarate was similar to that of fumarate (Fig. 6A). These data suggested that during the routine FFPE process, succinate and 2HG underwent similar extent of loss as glutamate, while fumarate underwent similar extent of loss as α-ketoglutarate.

### Comparison of oncometabolite profiles between paired FFPE and frozen tissues

**Figure 7:**
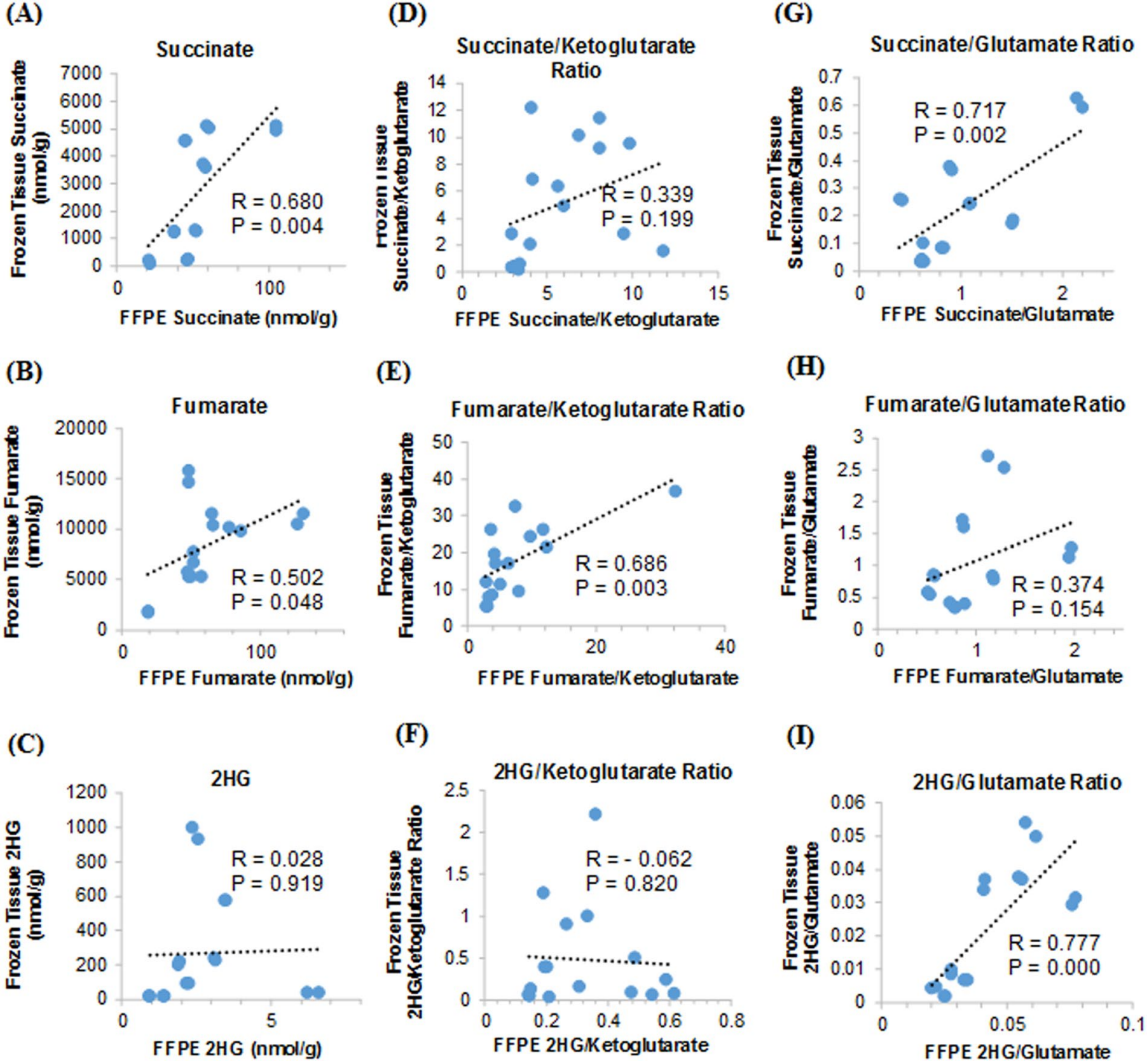
Comparison of oncometabolite profiles (as assessed by absolute oncometabolite tissue levels or the ratios of oncometabolites to α-ketoglutarate and to glutamate) between paired FFPE and frozen tissue specimens.

To validate the utility of FFPE specimens for oncometabolite profiling, correlations of oncometabolite profiles between FFPE and frozen tissue specimens needs to be demonstrated. We determined the concentrations of oncometabolites (succinate, fumarate and 2HG) and other TCA cycle metabolites in 8 paired FFPE and frozen tissue specimens. At average, FFPE specimens retained 2.7%, 0.8%, and 4.2% of succinate, fumarate, and 2HG levels, respectively, as compared to their respective levels in matched frozen tissues. There was a weak or no correlation in individual oncometabolite tissue levels between paired FFPE and frozen tissue specimens (Fig. 7A–C), likely due to inconsistent loss of oncometabolite during FFPE process among different specimens. However, significant correlations between FFPE and frozen tissues were found in succinate/glutamate ratio (R = 0.717, P = 0.002), fumarate/α-ketoglutarate ratio (R = 0.686, P = 0.003), and 2HG/glutamate ratio (R = 0.777, P = 0.000) (Fig. 7G,E,I). These data were well explained by the observation that during the routine FFPE process, succinate and 2HG underwent similar extent of loss as glutamate, while fumarate underwent similar extent of loss as α-ketoglutarate (Fig. 6A). Thus, normalization of individual oncometabolite concentrations to an endogenous internal standard (glutamate or α-ketoglutarate) could correct, in a large part, for sample-to-sample variations in the extent of oncometabolite loss during the routine FFPE process. Together with the evidence of a high correlation of individual oncometabolite tissue levels with the ratios of oncometabolite to α-ketoglutarate or to glutamate in frozen tissues (Fig. 5), these data collectively supported the use of the ratios of succinate to glutamate, fumarate to α-ketoglutarate, and 2HG to glutamate ratios in FFPE specimens as surrogate measurements for succinate, fumarate, and 2HG levels in FFPE specimens, respectively. In addition, given a high correlation between D- to L-2HG ratios and total 2HG or D-2HG levels in glioma FFPE specimens (Fig. 4B), D- to L-2HG ratios could also serve as the surrogate measurement for detection of elevated 2HG levels (Fig. 4B). We currently apply our method in several multi-center clinical studies to determine the ratios of succinate to glutamate, fumarate to α-ketoglutarate, 2HG to glutamate, and D- to L-2HG in FFPE specimens and explore their associations with tumor responsiveness to PARP inhibitors in patients.

## Materials and Methods

### Chemicals and reagents

Succinate, fumarate, 2-hydroxyglutarate (2HG), D- and L-α-hydroxyglutaric acid disodium salt, malic acid, α-ketoglutarate and glutamate monosodium salt were obtained from Sigma Aldrich (St. Louis, MO, USA). Stable isotope-labeled internal standards, including succinate-d6, fumarate-1,4-^13^C2, 2,3-d2, (RS)-2-hydroxyglutarate-2,3,3-d3, and 1,2,3,4-^13^C4 α-ketoglutarate were purchased from Cambridge Isotope Laboratories (Andover, MA, USA). The derivative reagent N-(p-toluenesulfonyl)-L-phenylalanyl chloride (TSPC) was purchased from VWR International (Radnor, PA, USA). All other chemicals and reagents were LC-MS grade.

### Instrumentation

All LC-MS/MS analyses were performed on an AB SCIEX (Foster City, CA) QTRAP 6500 system, which consists of a SHIMADZU (Kyoto, Japan) Nexera ultra high-performance liquid chromatography system coupled with a hybrid triple quadrupole and linear ion trap mass spectrometer. Analyst^®^ 1.6 software was used for system control and data acquisition, and MultiQuant 3.0 software was used for data processing and quantitation.

### Sample preparation

FFPE slides were deparaffinized by two washes in xylene (5 min each wash) in a glass slide staining dish (Fig. 1B). Residual xylene on the slide was dried at room temperature in a chemical hood, and the tissue on 3–5 FFPE slides of one sample was scraped into a pre-weighted Eppendorf tub and weighted. Deparaffinized FFPE tissue (0.5–1.5 mg recovered from 5 slides) or frozen tissue (1–10 mg) was homogenized in 200 µL water using a Precellys® homogenizer (at 2000 g for two 10 seconds with 5 seconds pause). Two aliquots of tissue homogenate were made: one for the determination of succinate, fumarate and total 2HG, and the other for the analysis of D- and L-2HG enantiomers.

For the determination of succinate, fumarate and total 2HG, 60 µl of tissue homogenate was spiked with the internal standard mixture of succinate-d6 (0.4 µM), fumarate-1,4-^13^C2 2,3-d2 (1 µM), and (RS)-2-hydroxyglutarate-2,3,3-d3 (0.2 µM), followed by protein precipitation twice with ice-cold methanol (240 μL) and 80% methanol (120 μL). The supernatant was combined and dried in a CentriVap Concentrator (Labconco, Kansas City, MO) at 10 °C. The residue was reconstituted in 60 µl of LC-MS grade water, and 5 µL of the supernatant was injected into the LC-MS/MS system.

For the analysis of D- and L-2HG enantiomers, 100 µl of tissue homogenate was spiked with the internal standard, (RS)-2-hydroxyglutarate-2,3,3-d3 (0.005 nmol), followed by protein precipitation twice with ice-cold methanol (400 μL) and 80% methanol (200 μL). The supernatant was combined and dried in a CentriVap Concentrator at 10 °C. The dried sample was subjected to the derivatization reaction by incubation with TSPC (2.5 mM in anhydrous acetonitrile) and anhydrous pyridine (2 µL) at 37 °C for 10 min with shaking, as described previously with modifications^17^. The reaction mixture was dried under a stream of nitrogen in a water bath (37 °C). The residue was reconstituted in 100 µL of 30% acetonitrile, and 5 µL of the supernatant was injected into the LC-MS/MS system.

### LC-MS/MS analysis of fumarate, succinate, and total 2HG

Chromatographic separation of oncometabolites (succinate, fumarate and 2HG) and other TCA cycle metabolites (α-ketoglutarate, glutamate, and malic acid) was achieved on a Phenomenex Synergi^TM^ Polar-RP column (150 × 2 mm, 4 µm) using a gradient elution consisting of mobile phase A (0.03% formic acid in water) and mobile phase B (0.03% formic acid in acetonitrile), at the flow rate of 0.25 mL/min. The gradient was programmed as: 0.3 min, 0% B; 5 min, 18.1% B; 7 min, 95% B; 7.5 min, 0% B; 13 min, 0%. The column oven was maintained at 35 °C.

To eliminate potential carryover, external and internal washes were implemented prior to and post the injection for both the auto-sampler syringe and injection port. The external wash was performed with 50% methanol (for R3) with 1 second rinse dip and 500 µL volume. The internal wash was performed at the sequence R1 to R0 to R2, with isopropanol – acetonitrile – methanol - water (1:1:1:1) for R1 and water with 0.03% FA for R0 and R2.

Column eluents were monitored under negative and positive electrospray ionization mode using the multiple reaction monitoring (MRM) on an AB Sciex QTRAP 6500 mass spectrometer. Mass spectrometric parameters (including ionization polarity, product ion, collision energy, declustering potential, and cell exit potential) were optimized to obtain the most sensitive and specific mass transitions for individual metabolites by direct infusion of the standard solutions into the ion source with a syringe pump. Succinate, fumarate, and 2HG were monitored at the mass transitions m/z, 117.0 > 73.0, 114.9 > 71.0, and 147.0 > 128.9, respectively. Their respective stable isotope-labeled internal standards, succinate-d6, fumarate-1,4-^13^C2, 2,3-d2 and (RS)-2-hydroxyglutarate-2,3,3-d3 were monitored at the mass transitions m/z, 121.0 > 76.9, 119.0 > 74.0, and 150.0 > 131.9, respectively. α-ketoglutarate, 1,2,3,4-^13^C4 α-ketoglutarate (internal standard), glutamate, and malic acid were monitored at the mass transitions m/z, 144.9 > 101.0, 149.0 > 104.9, 148.0 > 84.0, and 132.9 > 114.9, respectively. Other optimized mass spectrometric parameters were as follows: ion spray potential, −4500 V; nebulizer gas (GS1) and bath gas (GS2), 50 psi; curtain gas, 35 psi; collision gas, medium level; and source temperature, 475 °C. The dwell time for each MRM transition was 100 milliseconds.

### LC-MS/MS analysis of D- and L-2HG enantiomers

Chromatographic separation of TSPC derivatives of D- and L-2HG was achieved on a GL Sciences Inertsil-ODS3 column (2.1 × 250 mm, 5 µm) using a gradient elution consisting of mobile phase A (0.1% formic acid in water) and mobile phase B (acetonitrile/methanol, 50:50, v/v), at the flow rate of 0.2 mL/min, as described previously with modifications^17^. The gradient was programmed as: 3 min, 30% B; 8 min, 70% B; 20 min, 70% B; 21 min, 30% B; 30 min, 30%. The column oven was maintained at 40 °C.

Column eluents were monitored under negative electrospray ionization mode using the multiple reaction monitoring (MRM) on an AB Sciex QTRAP 6500 mass spectrometer. Mass spectrometric parameters (including ionization polarity, product ion, collision energy, declustering potential, and cell exit potential) were optimized to obtain the most sensitive and specific mass transitions for TSPC derivative of D/L-2HG by direct infusion of the standard solutions into the ion source with a syringe pump. TSPC derivatives of D/L-2HG, and the internal standard (TSPC derivative of (RS)-2-hydroxyglutarate-2,3,3-d3) were monitored at the mass transition *m/z*, 448.2 > 318.2 and 451.1 > 318.2, respectively. Other optimized mass spectrometric parameters were as follows: ion spray potential, −4500 V; nebulizer gas (GS1) and bath gas (GS2), 30 psi; curtain gas, 20 psi; collision gas, medium level; and source temperature, 500 °C. The dwell time for each MRM transition was 100 milliseconds.

### Method validation

The methods for the determination of succinate, fumarate and total 2HG, as well as D- and L-2HG enantiomers were fully validated using aqueous solution (LC-MS grade water) as well as pooled homogenates of FFPE and frozen tissues for the sensitivity, linearity, intra- and inter- accuracy and precision, recovery, and stability, based on the US Food and Drug Administration Guidance to Bioanalytical Method Validation.

Linearity, intra- and inter-day precision and accuracy were evaluated in aqueous solution. Stock solutions of succinate, fumarate, total 2HG, and D-/L-2HG were prepared in 50% methanol at a concentration of 10 mM, and stored in brown glass vials at – 80 °C. Calibrator and QC samples were prepared freshly on each day of analysis as serial dilutions in LC-MS grade water. Linearity was assessed at the concentration range of 0.02–10 µM, 0.2–100 µM, and 0.002–10 µM in water for succinate, fumarate, and total 2HG, respectively. For D- and L-2HG quantitation, calibrator and QC samples were subjected to the derivatization reaction, followed by LC-MS/MS analysis, as described above. Linearity was assessed at D- and L-2HG concentration range of 0.002–5 µM. Calibration curves were built by fitting the analyte concentrations versus the peak area ratios of the analyte to internal standard using linear regression analysis with the weighting of 1/x^2^ (where x represents the analyte concentration).

QC samples were prepared in water at the concentrations of 0.02 (LLOQ), 0.06, 1.2, and 6.0 µM for succinate; 0.2 (LLQQ), 0.6, 12.0, and 60 µM for fumarate; 0.002 (LLOQ), 0.006, 1.2, and 6 µM for total 2HG; and 0.002 (LLOQ), 0.006, 0.8, and 4.0 µM for D-/L-2HG. Intra- and inter-day precision and accuracy were assessed for the calibrator standards (each in duplicate) and QCs (including LLOQ, low, medium, and high QCs, each in quintuplicate) on three days. The accuracy was assessed as the relative estimation error of the determined concentration to nominal concentration. The intra- and inter-day precisions were estimated by one-way analysis of variance (ANOVA) using the JMP^TM^ statistical discovery software version 5 (SAS Institute, Cary, NC, USA), as described previously by us^21^.

The recovery of added analyte was assessed by spiking individual oncometabolites at low (i.e., ~30% of endogenous level) and high concentrations (i.e., high QC levels) into pooled homogenate of FFPE or frozen tissue (n = 6). The recovery was calculated as (final concentration − endogenous concentration)/added concentration.

The matrix effects of FFPE or frozen tissue homogenates on individual oncometabolites were assessed by the matrix factors of their respective isotope labeled internal standard. The matrix factor was determined as the ratio of the peak area of an internal standard in post-extracted matrix solution to that in aqueous solution.

Stability tests of succinate, fumarate, and 2HG were performed to evaluate the bench-top stability in water (at 1 and 100 µM) and in pooled homogenate of FFPE or frozen tissue, autosampler stability in water and post-extracted solution, and freeze-thaw stability in pooled homogenate of FFPE or frozen tissue.

### Determination of oncometabolite concentrations in human FFPE and frozen tissue specimens

Human renal cell carcinoma and normal kidney frozen tissue and FFPE specimens were obtained from the Genitourinary Biospecimen Repository at Yale University. All tumor samples were deidentified. The sample bank was approved by the Yale Human Investigation Committee (HIC), and all subjects provided informed consent. There are no identifiable images presented. All procedures were in accordance with the ethical standards of the responsible committee on human experimentation or with the Helsinki Declaration of 1975 (as revised in 1983).

Frozen and FFPE tissue specimens were processes and subjected to LC-MS/MS analyses for quantitation of oncometabolites and TCA cycle metabolites, as described above. Since individual metabolite levels varied significantly across different samples, both original homogenate and 20-fold diluted homogenate samples were processed and subjected to LC-MS/MS analyses to ensure the measured concentrations to fall within the respective linear calibration curve range.

### Evaluation of oncometabolite loss during FFPE process

To minimize the impact of tissue heterogeneity on metabolite concentration measurements, a piece of rat liver tissue (~500 mg) was cut into 12 sections, and alternating sections were grouped into 4 groups to evaluate the loss of metabolites during each step of FFPE process (Supplementary Fig. 1). Group 1 (Section # 3, 7, 11) was used for determination of oncometabolite concentrations in frozen tissues as the control. Group 2 (Sample # 2, 6, 10), Group 3 (Sample # 4, 8, 12), and Group 4 (Sample # 1, 5, 9) were used to assess the preservation of oncometabolites after formalin fixation, formalin fixation plus ethanol dehydration, and formalin fixation plus ethanol dehydration followed by toluene, respectively. The standard FFPE procedure was applied. Specifically, tissue sections were fixed in 10% neutral buffered formalin (with the volume 15–20 times higher than specimen volume) for 6 hours, and dehydrated in 3 ethanol baths with increasing concentrations of ethanol (70%, overnight; 85%, 0.5 h; 90%, 0.5 h), followed by 3 toluene baths (0.5 hours each) to replace ethanol trapped inside tissues. After each step, tissue sections were weighted. Metabolite concentrations were determined in tissue sections and the solutions used in the fixation and dehydration process.

### Statistical analysis

Correlations were examined by Pearson Correlation analysis. P values were two-sided, and P < 0.05 was considered as statistical significance. Statistical analyses were performed with the IBM SPSS Statistics 25 software.

## Supplementary information

Supplementary Materials

## Data Availability

The data that support the findings of this study are available from the corresponding author upon reasonable request.
