# Supplementary material for: Quantitative Profiling of Oncometabolites in Frozen and Formalin-Fixed Paraffin-Embedded Tissue Specimens by Liquid Chromatography Coupled with Tandem Mass Spectrometry
Source: Sci Rep. 2019 Aug 2;9:11238. doi: 10.1038/s41598-019-47669-5 (PMC6677826; doi:10.1038/s41598-019-47669-5)
Supplement: Supplementary file 1 — Supplementary Materials [file 41598_2019_47669_MOESM1_ESM.pdf]

(Supplementary Materials)

Quantitative Profiling of Oncometabolites in Frozen and Formalin-Fixed Paraffin-Embedded Tissue Specimens by Liquid Chromatography Coupled with Tandem Mass Spectrometry

Xun Bao[1‡], Jianmei Wu[1‡], Brian Shuch[2,3], Patricia LoRusso[2], Ranjit S. Bindra[2*], and Jing Li[1]*

[1] *Karmanos Cancer Institute, Wayne State University School of Medicine, Detroit, MI 48201*

[2] *Yale Cancer Center, Yale University School of Medicine, New Haven, CT 06520*

[3] *Department of Urology, David Geffen School of Medicine at UCLA, Los Angeles, CA, 90049*

Supplementary Table 1 Mass transitions and optimized mass parameters for individual oncometabolties, other TCA cycle metabolites, and their respective isotope labeled internal standards.

Q1	Q3	Metabolite name	DP	CE	CXP
Positive ionization mode					
148	84	Glutamic acid	35	21	12
Negative ionization mode					
117	73	Succinic acid	-42	-16	-7
114.9	71	Fumaric acid	-40	-12	-9
132.9	114.9	Malic acid	-30	-15	-12
144.9	101	α-ketoglutaric acid	-35	-12	-12
147	128.9	2-Hydroxyglutaric acid	-70	-15	-12
149	104.9	$^{13}C_4$ α-ketoglutarate	-40	-10.6	-14
121	76.9	D_6-Succinic acid	-31	-18	-9
119	74	$^{14}C_2$_D_2_Fumaric acid	-33	-10	-8.4
150	131.9	ISO-DL-2-HG	-35	-15	-12
448.2	318.2	TSPC-D-2-HG	-32.09	-18.07	-26.35
448.2	318.1	TSPC-L-2-HG	-32.09	-18.07	-26.35
451.1	318.2	ISO D-2-HG	-50	-20	-23
451.1	318.1	ISO L-2-HG	-50	-20	-23

Supplementary Table 2 Intra- and inter-day precision and accuracy for the calibrator standards of succinate, fumarate, and 2-HG, prepared in LC-MS grade water [a]

Metabolite	Nominal concentration (µM)	Determined concentration (µM)	Average accuracy[c] (%)	Intra-precision (%)	Inter-Precision (%)
Succinate	0.02 (LLOQ)	0.020 ± 0.002	4.8	0.8	7.5
	0.05	0.046 ± 0.003	-8.6	2.2	6.6
	0.1	0.10 ± 0.01	-4.4	3.0	10.5
	0.2	0.19 ± 0.02	-5.4	9.5	3.2
	0.5	0.47 ± 0.02	-5.6	2.1	4.5
	1.0	1.01 ± 0.05	0.8	5.4	- [b]
	2.0	2.05 ± 0.07	2.3	4.6	- [b]
	5.0	5.23 ± 0.25	4.5	2.2	4.8
	10.0	10.49 ± 0.40	4.9	1.3	4.0
Fumarate	0.2 (LLOQ)	0.20 ± 0.01	1.3	3.0	2.1
	0.5	0.50 ± 0.03	0.6	7.6	- [b]
	1.0	0.98 ± 0.09	-0.7	9.3	- b
	2.0	2.04 ± 0.12	4.5	4.8	3.5
	5.0	4.80 ± 0.29	-5.4	7.2	- b
	10.0	9.85 ± 0.45	-2.2	4.8	- b
	20.0	20.60 ± 0.98	2.0	5.0	- b
	50.0	50.81 ± 1.62	0.2	3.0	1.3
	100.0	101.43 ± 3.54	0.8	4.1	- b
2-HG	0.002 (LLOQ)	0.0020 ± 0.0002	-0.6	12.4	- b
	0.005	0.0049 ± 0.0006	-2.3	12.7	3.8
	0.01	0.0098 ± 0.0060	-2.0	6.9	- b
	0.02	0.020 ± 0.001	1.7	5.8	- b
	0.05	0.051 ± 0.002	1.1	5.0	- b
	0.1	0.10 ± 0.01	-0.2	8.6	- b
	0.2	0.21 ± 0.01	3.8	5.8	- b
	0.5	0.51 ± 0.03	2.2	5.4	- b
	1.0	1.03 ± 0.02	3.5	2.3	0.7
	2.0	2.09 ± 0.10	4.7	3.6	3.4
	5.0	4.99 ± 0.13	-0.2	2.2	2.1
	10.0	9.54 ± 0.48	-4.6	5.0	- b

[a] Each calibration curve was evaluated in duplicate on three different days.
[b] No additional variation was observed as a result of performing assay in different days.
[c] Accuracy was assessed as the relative estimation error of the determined concentration to nominal concentration.

Supplementary Table 3 Intra- and inter-day precision and accuracy for the calibrator standards of D- and L-2HG, prepared in water after derivatization reaction[a]

Nominal concentration (μM)	Determined concentration (μM)	Average accuracy[c] (%)	Intra- precision (%)	Inter- precision (%)
**D-2HG**				
0.002 (LLOQ)	0.0021± 0.0003	-2.5	14.5	-[b]
0.005	0.0047 ± 0.0003	-7.0	5.9	2.2
0.01	0.0099 ± 0.0007	-1.3	5.5	5.1
0.02	0.0187 ± 0.0008	-6.7	2.7	4.1
0.05	0.0499 ± 0.0044	-0.3	9.6	-[b]
0.1	0.1002 ± 0.0055	0.2	7.1	-[b]
0.2	0.2093 ± 0.0109	4.7	4.9	2.0
0.5	0.5204 ± 0.0207	4.1	4.5	-[b]
1	1.0192 ± 0.0245	1.9	3.1	-[b]
2	2.0646 ± 0.0654	3.2	0.9	3.4
5	4.8768 ± 0.2392	-2.5	3.9	3.3
**L-2HG**				
0.002 (LLOQ)	0.0020± 0.0001	0.8	7.3	-[b]
0.005	0.0046 ± 0.0003	-8.7	2.8	7.0
0.01	0.0101± 0.0006	1.3	6.6	-[b]
0.02	0.0201 ± 0.0011	0.3	5.9	-[b]
0.05	0.0505 ± 0.0039	0.1	6.0	5.4
0.1	0.0993 ± 0.0055	-0.7	7.1	-[b]
0.2	0.2058 ± 0.0092	2.9	2.7	4.0
0.5	0.4996 ± 0.0274	-0.1	6.9	-[b]
1	1.0274 ± 0.0457	2.7	0.5	4.9
2	1.9703 ± 0.1166	-1.5	6.4	-[b]
5	4.8857 ± 0.3034	-2.3	4.8	4.4

[a] Each calibrator standard was evaluated in duplicate on three different days.
[b] One-way analysis of variance: No additional variation was observed as a result of performing assay in different days.
[c] Accuracy was assessed as the relative estimation error of the determined concentration to nominal concentration.

Supplementary Table 4 Intra- and inter-day precision and accuracy for the calibrator standards of individual other TCA cycle metabolites prepared in LC-MS grade water.

Nominal concentration (µM)	Determined concentration[a] (µM)	Average accuracy[c] (%)	Intra-day precision (%)	Inter-day precision (%)
**Glutamate**				
0.02 (LLOQ)	0.020 ± 0.002	0.5	13.8	-[b]
0.1	0.108 ± 0.008	8.1	8.8	-[b]
0.2	0.216 ± 0.008	7.9	2.4	3.2
0.5	0.500 ± 0.030	-0.1	5.6	2.1
1	0.933 ± 0.075	-6.7	5.5	6.5
2	1.782 ± 0.044	-11.1	1.2	2.6
**Malic acid**				
0.02 (LLOQ)	0.021 ± 0.002	4.0	13.5	-[b]
0.1	0.100 ± 0.008	0.4	9.5	-[b]
0.5	0.507 ± 0.021	1.4	5.2	-[b]
1	1.021 ± 0.042	2.1	2.6	3.5
5	5.309 ± 0.397	6.2	6.8	3.5
10	9.760 ± 0.876	-2.4	9.1	-[b]
**α-ketoglutarate**				
0.2 (LLOQ)	0.21 ± 0.01	3.4	4.8	-[b]
0.5	0.50 ± 0.03	-0.5	9.0	-[b]
1	0.94 ± 0.04	-5.9	4.2	1.1
5	5.04 ± 0.29	0.8	5.4	2.0
20	21.34 ± 0.89	6.7	2.6	3.7
100	95.41 ± 3.78	-5.6	5.1	-[b]

[a] Each calibrator standard was evaluated in duplicate on three different days.
[b] One-way analysis of variance: No additional variation was observed as a result of performing assay in different days.
[c] Accuracy was assessed as the relative estimation error of the determined concentration to nominal concentration.

Supplementary Table 5 Intra- and inter-day precision for glutamate, malic acid, and α-Ketoglutarate in pooled FFPE and frozen tissue samples.

	Determined concentration[a] (μM)	Intra-day precision (%)	Inter-day Precision (%)
**Glutamate**			
FFPE	0.551 ± 0.049	4.7	9.1
Frozen tissue	139.684 ± 4.580	2.0	3.0
**Malic acid**			
FFPE	0.286 ± 0.029	8.1	7.3
Frozen tissue	100.156 ± 13.969	7.3	13.7
**α-Ketoglutarate**			
FFPE	0.379 ± 0.027	7.3	-[b]
Frozen tissue	10.679 ± 0.815	8.7	-[b]

[a] Endogenous levels of glutamate, malic acid, and α-Ketoglutarate were measured in pooled homogenates of FFPE or frozen tissues on 3 days, with 5 replicates on each day.

[b] One-way analysis of variance: no additional variation was observed as a result of performing assay in different days.

Supplementary Figure 1 Schematic illustration of experiment procedure for evaluation of metabolite loss during the formalin fixation and ethanol dehydration process.

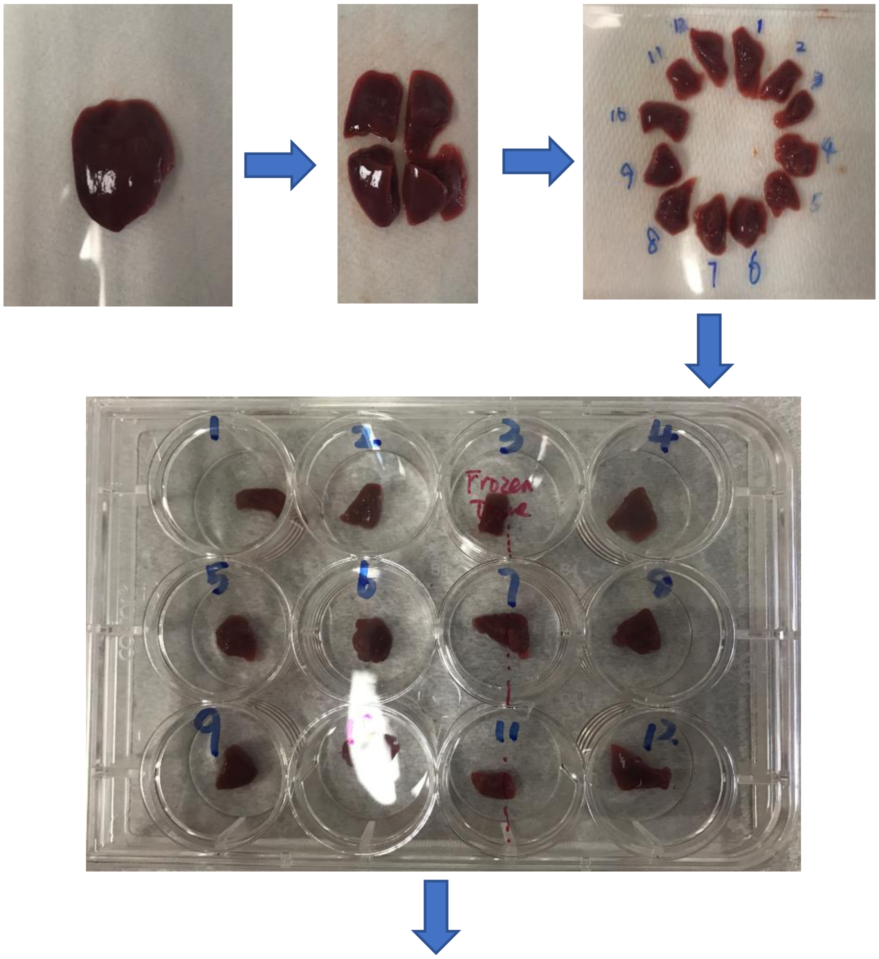

Group 1 (Section # 3, 7, 11): Frozen tissues
Group 2 (Section # 2, 6, 10): Subjected to formalin fixation
Group 3 (Section # 4, 8, 12): Subjected to formalin fixation and dehydration in ethanol
Group 4 (Section # 1, 5, 9): Subjected to formalin fixation and dehydration in ethanol followed by toluene
